# ‘It wasn’t what I was suited for’: regretful mothers negotiating their reproductive decision and mother role

**DOI:** 10.1136/medhum-2023-012717

**Published:** 2023-11-17

**Authors:** Maja Bodin

**Affiliations:** 1 Centre for Medical Humanities, Uppsala University Department of History of Science and Ideas, Uppsala, Sweden

**Keywords:** family planning, reproductive medicine, Gender studies

## Abstract

This study contributes to our understanding of why women without a longing to have children and who, in theory, have the possibility of refraining from parenthood still become mothers. The article is based on in-depth interviews with six Swedish mothers who never longed to have children in the first place. It illustrates how they make sense of their reproductive decision-making process and their current role as a mother. The analysis shows how reproductive decision-making is highly influenced by cultural perceptions of proper womanhood and the idea that every woman has an innate longing to have children, as well as other people’s wishes and pressure. Although the mothers did their best to align with motherhood expectations, their narratives show that they are still oriented towards non-motherhood on an emotional level. This manifests through their experiences of existential regret about having children. Hence, the mothers’ understandings of their path to motherhood reveal a complex conflict between outer expectations and inner wishes, which destabilises the idea of reproduction as a promise of happiness.

I love my children now and can't really imagine being without them, but I still sometimes struggle with the mother role. It really doesn't feel like I'm meant to be a mom.

This quote comes from one of the Swedish mothers that I interviewed in my project on parenthood regret. She is one of six mothers who never really wanted to have children in the first place, and today regrets her decision to have them. Not being oriented towards motherhood is quite uncommon in the Swedish context, where only 13.5% of women remain childless (voluntary or involuntary) at age 45 years (Statistics Sweden 2020). Compared with many other European countries, which have seen a steady decline in childbearing, Sweden has had a stable fertility rate of just under two children per woman in recent decades. This is likely a result of family friendly policies that have been in place since the 1970s to enable parents to combine family life and work life (Esping-Andersen 2016). In combination with high levels of gender equality, and strong reproductive rights, Sweden appears as the perfect place to raise the family of one’s choice.

I therefore find these six women particularly interesting, especially in terms of regret, since they put regret (as a phenomenon/concept) in a different light. According to literature on psychology, regret is based on the assumptions that one has (1) Made a deliberate choice, (2) That the choice was made with the best intentions in mind (positive expectations), and (3) One realises in hindsight that another choice would have been better (Dijk and Zeelenberg 2005). Quite rightly, previous studies on parenthood regret have shown that regret is often related to unfulfilled expectations of parenthood (Heffernan and Stone 2021; Matley 2020; Moore and Abetz 2019; Sihto and Mustosmäki 2021). According to these studies, regret usually arises when it turns out to be much more difficult than expected to care for children and live up to (sometimes unrealistic) parenthood ideals. The experience is hence evaluated as being not worth the sacrifices.

But the mothers I have chosen to focus on in this article were pessimistic about motherhood from the start. They had been ‘oriented towards non-motherhood’ (Donath 2015, 205) from an early age and assumed before having children that motherhood was not right for them (at least that is what they shared in retrospect). Their stories raise numerous questions. If they did not have positive expectations on motherhood, is it still regret that we are talking about? How come women without a longing for children, who live in a society strongly advocating freedom of (reproductive) choice, still become mothers? Particularly in a secular context with gender equality ideals and access to contraceptives and free abortion, we might think that involuntary mothers should not exist anymore. Are reproductive choices as free as they tend to be portrayed? These are some of the questions that I will explore in this article.

## The holy motherhood

The norm that women should have children appears to be almost universal historically and culturally. In Western societies, motherhood has long been at the core of religiously sanctioned norms regarding womanhood and family. In the course of secularisation, these have not disappeared; rather, they have been absorbed by modern values. Maternal behaviours are dependent on the economic and social circumstances of motherhood, values regarding women and the status of the child (Badinter 1981; Eriksen 2017). Each era and culture has its own rules regarding proper maternal behaviour and feelings, as well as explanatory models for those expected feelings and behaviours. In contemporary Western culture, parents are expected to practise close, intensive and risk-oriented parenthood and put their children’s well-being first. This applies even before the child is born, or for that matter conceived (Waggoner 2013).

In the specific Swedish context, the understanding of motherhood stems from a Lutheran system of interpretation, in which it is seen as part of a woman’s God-given nature (Westberg 2016). During the twentieth century, moral happiness, medical and psychological discourses about motherhood have replaced the explicit religious content in this idea, while maintaining and even reinforcing the notion of motherhood as ‘natural’. Westberg shows, for example, how *hormones* have become a central explanatory framework for proper motherhood. Kling has also described how Swedish women who had difficulties conceiving in the 1930s–1940s believed that there was something missing in their bodies, such as hormones, which made them infertile and thereby deviant as women (Kling 2010). This logic is also apparent in recent studies on childless women, who tend to reason that it is probably a lack of hormones that has caused them to not long for children, nor feel the maternal instinct (Allen and Wiles 2013; Bhambhani and Inbanathan 2018; Carmichael and Whittaker 2007; Peterson and Engwall 2013). If hormonal imbalance is thought of as the underlying reason for childlessness, it also implies that it is something that can be fixed through medical or psychological interventions.

Hence, not only are practices governed by a system of rules and expectations, so too are emotions. Arlie Hochschild describes how each culture has its own set of ‘feeling rules’ Hochschild (2012). These come to consciousness as moral injunctions; what we should or should not feel, and what we have a right or no right to feel. Wanting children, feeling happy about parenthood and love for your child are seen as natural—we assume ‘nature’ to do the work for us. Parenthood is, therefore, also expected to need less guidance and emotion work. In reality, however, parents and children feel both love and hate for each other, but ambivalent feelings in relation to parenthood tend to fall outside most modern Western cultural scripts. Because of this, Hochschild argues, parenthood in fact requires quite a lot of emotional work to manage socially unacceptable feelings, such as regret.

## Regret about reproductive decisions

Regret has been described as one of our most common emotions (Buchanan *et al*. 2016), and it is related to counterfactual thoughts about how life could have been if we had chosen otherwise (Dijk and Zeelenberg 2005). Women report more regrets than men, and their regrets more often concern romantic relationships, family relationships and parenting (Dijkstra and Barelds 2008; Roese and Summerville 2005). Given that these are common areas of regret for women, it is interesting to notice that few things are as taboo as regretting motherhood. Donath (2015) has argued that this cultural taboo is due to four intersecting cultural logics: (1) That motherhood is seen as natural and women’s reason for existence, (2) It is worth it no matter the costs, (3) Our cultural ‘feeling rules’ (Hochschild 2012) do not allow women to feel anything else than positive feelings towards motherhood, and (4) Motherhood is framed as a promise. Even if we initially are neutral or negative about having children, our temporal logic of ‘linear progress over time’ still leads us to think that positive feelings towards motherhood will develop. Queer scholar Sarah Ahmed argues along the same lines that family life is culturally framed as an object of happiness, something that we believe will influence us in the best way possible (Ahmed 2010). The promise of happiness suggests that happiness lies in front of us if we do the right things, such as starting a family with children.

Researchers have claimed that the feeling of regret arises if we blame ourselves for a negative outcome, and feel that we could have *chosen* differently. If we instead think that other people or the circumstances have caused the negative outcome, the dominant feeling is usually disappointment rather than regret. What is problematic about these quantitative studies of regret is that they tend to simplify the decision-making process. Donath (2015) highlights in her study of regretful mothers that when it comes to reproductive decision-making, the binary categorisation of choice/no choice is insufficient to understand the process. It hides things like uncertainty, contradictions and randomness, as well as social forces and power relations at play. Shaw (2011), who interviewed voluntary childless women, also disputes the binary of choice/no choice in reproductive decision-making, referring to how choices are rarely as consistent in reality, as in theory. She argues that we should instead understand the accounts of reproductive decision-making as demonstrations of the historicity of one’s choice: how choices are contingent on time, on one’s personal history, the times through which one has lived and the relationships one has had.

Donath further argues that more attention should be paid to notions of will, desire, orientations and consent. By orientations, Donath refers to ‘a woman’s self-perception of her emotional stance (the integration of thoughts, feelings, desires and fantasies) regarding the idea of having children and raising them’ (Donath 2015, 205). Her orientation may be inconsistent and change over time. Orientations are also an important theoretical concept in queer theory, where being oriented is described as being turned towards specific, recognisable or familiar objects that help us find our way in the world (Ahmed 2006). When we orient ourselves towards something and follow a certain path, we invest in it socially and expect something in return; namely that it will get us somewhere. Ahmed argues that we turn towards objects that are reachable and bodily available to us, and we are in line with others when we face the direction that is already faced by others. And so, bodies get directed in some ways more than others, such as towards heterosexual relationships and parenthood.

Another aspect of regret is that it is often framed as the result of a mistake. In her study on regret in relation to abortion, Madeira (2014) claims that it is wrong to expect that regret about a reproductive choice is a result of flawed decision-making. Instead, regret more likely signifies a woman’s deep reflection on the decision, and the value she places on her primary relationships. Madeira suggests that we should understand reproductive decisions as *relational autonomy*, where regret can signify a creative, thoughtful, considered, reflexive and subjective exercise (2014, 39–40). The relational autonomy model suggests that autonomy is always partial, and choice is always constrained to some extent. The model enhances our understandings of how emotions are mediated by social relations and that a woman’s feelings towards parenthood can change over time. Inspired by these previous studies on reproductive decision-making, I will analyse the accounts of mothers who had children reluctantly and now regret having them, specifically focusing on their orientations (Ahmed 2006; Donath 2015), historicity and relationality (Madeira 2014; Shaw 2011).

## Material and methods

This article is part of a larger project on parenthood regret, in which I interviewed 29 parents between September 2021 and May 2022. I conducted the interviews face to face in the participants’ home or workplace, or via video communication online. To be included in the study, the parents had to have a persistent feeling of regretting parenthood, a child that was at least 18 months old, and not be suffering from postpartum depression or any other severe mental health issue. Participants were recruited through an advertisement in social media and other social networks, and word of mouth. Some also contacted me after hearing or reading about the study on the radio or in newspapers. A total of 46 parents contacted me to get more information about the study. Everyone was informed about the inclusion criteria and that participation was voluntary, that they could withdraw from the study at any time without giving a reason, that their participation would be treated with confidentiality and that the study had been approved by the national ethical review agency (Dnr 2020–05957). In the end, 25 mothers and 4 fathers agreed to participate in the study and were interviewed during the study period (which was limited by time). I would say that saturation was reached among younger mothers, but not among older mothers or fathers.

The interview started with a few questions about the participant’s background, family situation and motives to participate in the study. Thereafter, I posed one core question which was ‘Can you tell me about your experiences of regretting parenthood?’. Thereafter, the interview was unstructured and flowed like a conversation where the participants could decide what to share and I asked follow-up questions. The interviews usually lasted between 40 min and 90 min. All interviews were audio-recorded and transcribed verbatim. During transcription, all sensitive personal information was removed and names replaced with pseudonyms.

For this particular article, six interviews were chosen for closer examination, based on their intriguing commonality: women who talked about becoming mothers without really wanting to. This communality became clear to me after having read all interviews and making an initial thematic coding. Five of these particular mothers were aged 33–40 years at the time of interview and had small children (18 months to 6 years). The sixth woman, whom I have called Kerstin, stands out as she was in her 80s and had adult children, as well as grandchildren. Apart from this age difference, the total sample is very homogenous, something that is usually strived for in studies using interpretative phenomenological analysis (IPA): I interpret the women as white and lower-to-upper middle-class. Most of them had a university education and had (had) gainful employment. They expressed gender equality ideals and had used the public day care services to be able to go back to work after parental leave. All women had had children in an heterosexual relationship. Three of them were currently married to and living with the father of their children. The other three had separated from the father, whereof two had joint physical custody of their children. One had sole custody since the father of the child had been violent. Those who had separated had been unhappy with the division of labour in the home, and felt that their partner had not given them enough practical or emotional support during a difficult time as a new mother. The ones who were still in a relationship had discussed these equality issues in-depth with their partner, and the partner had assumed more responsibility for the children.

The analytical process was inspired by IPA, which is a qualitative research approach used to examine how people make sense of major life experiences (Smith, Flowers, and Larkin 2009). One could say that the researcher is trying to make sense of the participant trying to make sense of what is happening to them. IPA is especially valuable when examining topics which are complex, ambiguous and emotionally laden, like reproductive decision-making and parenthood. The analysis is interpretative and inductive, and the researcher moves from the particular to the shared, and from the descriptive to the interpretative, in a cyclic way. One starts with analysing single cases, and then moves on to look for patterns across multiple cases.

The process is also self-reflexive, and I used field notes during data collection that I later came back to during the text analysis. I acknowledge that my study was part of framing and labelling the participants’ experiences in terms of regret, in that the study advertisement and the interview put feelings into words and labels on emotions that had previously not been defined. In several cases, the participant told me during the interview that it was the first time they shared their feelings of regret and put words to how they felt. Because of this, and the sensitivity of the topic, participants were offered to check quotes before publication.

## Findings

The women in this article felt immediate regret about becoming a parent, knowing from the start that they should not have had children and that they had done it against their inner wish. Each of them expressed this in a strikingly similar manner during the very first minutes of the interview:

Amanda: I was totally convinced that I would not have children; it really wasn’t something that felt relevant.

Frida: As long as I can remember, I have always felt that having children is not something for me. I have never wanted to have children, ever.

Jonna: I am a person who never wanted to have children. I have never dreamt of having children or longed for it or felt the biological clock that people talk about.

Kerstin: I have never felt the longing for children which is talked about. Never.

Rebecca: I have never in my life felt a longing to have children, any desire to do so.

Wilma: I have never in my whole life wanted to have children as far as I can remember.

By expressing that they did not feel a longing for children, these women reveal that longing for children is what is expected by women, and that they deviate from this norm. After having said this, they developed or justified their reluctance to become a mother more thoroughly. Through their explanations, they firmly grounded their orientation towards non-motherhood in such a way that they did not desire or fantasise about having children and raising them (Donath 2015). Instead of motherhood, they had been fantasising about an intellectual life, a life of hobbies or of romance. They returned to childhood memories and talked about how they never really got the point of playing with dolls or baby-sitting when they were young. Some expressed that their unwillingness to become a mother was related to fear or unease. They talked about being afraid of giving birth or of becoming a single mom, of feeling uncomfortable with children and not knowing how to behave around them. In this way, they made motherhood into an object of uncertainty, rather than something natural and purely an object of happiness (Ahmed (2010)). The participants also talked about how they knew that children limit one’s life and one’s flexibility, and that they had not been interested in sacrificing their freedom for the sake of motherhood. Many of these explanations are similar to how child-free women talk about their reproductive decision-making process (Peterson and Engwall 2013; Shaw 2011) but also to some involuntary childless women (Rondung, Magnusson, and Ternström 2022). This confirms how reproductive choices are much more complex than what binary categories such as choice/no choice or voluntary/involuntary allow us to understand (Donath 2015; Letherby 2002). Rather than a binary choice, reproductive decision-making is a process where decisions emerge and are woven into a culturally, socially and psychologically coded narrative.

## Not longing is not reason enough

The women in the study were thus initially not oriented towards motherhood. Why then did they become mothers? Their narratives take us back to the myths of womanhood and what is seen as natural. Women are expected to long for parenthood, and their bodies are expected to follow their natural purpose. A woman who *can* have children *should* also have them. One could thus say that the women turn towards motherhood, even without a longing, because it is reachable and bodily available to them (Ahmed 2006). Kerstin, who is now in her 80s, explains how ‘not longing’ was disqualified as an acceptable reason to not have children when she was young:

My parents thought it was a bit strange that the years passed, and we never had children. I was around 30 at the time. I just said no, I’m not interested. But my husband started to get a bit worried about the birth control pills, since we started to get reports about side effects, and he thought that I should go to a gynaecologist to get examined. And the gynaecologist was horrified by how things looked down there and said that “I don’t think you will be able to have children”. Thank God, I thought, that’s a relief, then I have a reason to avoid it. But she did not think so, no she did not. I said that I was not that interested in having children, but she said “Well, you will regret that, so I think we should start medication”. I had nothing to say against it, other than that I didn't feel like it. Yes, so I started the medication.

Kerstin and her husband have talked a lot about what happened and agreed that it was mostly his fault, since he insisted that she should stop using birth control, but she was responsible since she agreed to stop using them and instead started taking the medication that would improve her fertility. Her narrative also reveals trust in medical authority; the gynaecologist’s words that they probably could not have children and her recommendation to start medication.

Just like Kerstin’s gynaecologist, Rebecca also mentioned the issue of possible future regret for *not* having children. She felt heavily affected by this possible regret, pronatalist messages in society, and a fear of missing out. Again, we can see how the family is constructed as a promise of happiness:

Everyone who has children says that you never regret it no matter how difficult it gets; it is never the wrong decision. Because it is so fantastic to have children and so special. And it gives life more meaning, and when you finally have children, they become the meaning of life. And when I heard that, I got insecure of my own feelings and started to think that maybe I just don’t realise what it could mean to have children because I’m not used to spending time with children. Maybe if we had children, I would be struck by love and think “God, what if I had missed this”, and maybe if I didn’t have children, I would regret it when it was too late. Because that is also it; you can’t just wait until you start to long for it. You have to make a decision before the body says no. As a woman, you have that to think about as well; you have to make a decision quite fast, when you get to 35.

Rebecca and her husband, who did not long much for children either, finally made ‘an intellectual decision’ to have children rather than an emotional, based on the truths that had been presented to them about parenthood and female fertility.

Wilma had quite a different motive to why she had a child without really wanting to, but she also mentioned fertility stress as a contributing factor and a fear of not belonging. She fell in love with a man who already had children, and was happy at first about being a stepmother. However, after a while, she started to feel that she was not a true member of the family, and began to think that the only way in was to have a biological child with her husband. She felt that she needed to make a decision quickly before her fertility declined, and she clearly remembers the inner dialogue she had with herself about her motives:

On my way to work one morning, I thought, Wilma, are you really doing the right thing? Are you doing this for the right reason? What if you regret it afterwards? Because now you are about to have a child to belong, not because you want one. I had that thought, but it was difficult to accept it so I pushed it away // The birth of the child was very traumatic, and I went to a psychologist afterwards to process it. But when time had passed and everything had calmed down, it was still there. I thought during the first year that I regretted it because of the traumatic birth. But the feeling is still alive. // Inside of me, there is this thought that popped up on my way to work (several years ago), because it became the truth. I love my child, but you can have two feelings at the same time. You can love the human you have created – it is not the person you regret – but I regret that I became a parent. And I have really become grounded in the feeling that I have had my whole life; I really do not want to become a parent.

Although already being a mother, Wilma used the future tense here (not wanting to become) which confirms that she is still oriented towards non-motherhood. But she is also very clear that it is not a lack of love for her child that is the problem. What she regrets is that she went against her inner wish, and that she did not deal with her issues (the feeling of not belonging) in a constructive way, like going to couples’ therapy, before making the decision to become pregnant.

## Freedom of choice?

Parallel to the rational and scientific arguments, reproductive decisions were negotiated in relation to emotions, relationships and social norms. Amanda talked about how her ambivalent feelings of not really wanting to have children, but at the same time trying to fit the model of a normal woman, made her finally leave the decision to faith. Her partner wanted to have children, and one day while having sex, she decided to not protect herself from pregnancy. If she—which she believed was highly unlikely—would become pregnant this single time, it was meant to happen. And so, it happened. Since she had made this decision to ‘gamble’, she felt responsible for what had happened, and therefore did not feel morally entitled to having an abortion.

Frida tells a somewhat similar story about her reproductive decision-making process. She had told everyone she knew that she was never going to have children, including her partner, when they first met. He, on the other hand, had been very keen on becoming a father. Frida thought that she could get out of the situation by saying that she would not have children unless he put a ring on her finger, thinking that it would never happen. But several years later, he proposed and they got married, which made Frida feel terribly guilty:

I wanted to be with him, and I knew he didn't want to get married but it was like I had led him to get married, so I got the feeling that I owed it to him to give him children now because he gave me my dream wedding. But then I talked a lot with friends and colleagues, and because I had passed 30 and had been on birth control for over a decade, there were so many people around me who said "God, having children after 30, that’s impossible, it’s going to take so long! It’s not even sure you can get pregnant now that you've been on birth control for so long” and stuff like that. Then I felt a bit like this; hm, we might not even be able to have a baby! So, then I thought it doesn't really matter; then I can stop using contraceptives, because the chance of getting pregnant is so small. I thought. And a month later, I was pregnant.

Although many generations apart, Frida describes how she put faith in the same kind of misconception around birth control that Kerstin had done in the 1970s, namely that hormonal contraceptives reduce fertility. And just like Kerstin, she became pregnant soon after she stopped using them, which was quite a shock. She continues:

I started pretending like I wasn't pregnant, to cope. It’s so hard to explain, but I didn't want to be pregnant. I didn't want to have children, but he was so happy about it. The first twelve weeks are always a bit critical, and I used tobacco at that time and it took me two or three weeks before I stopped because I was like this “If there is a miscarriage, it’s not my fault”. It was almost that I hoped it would be a miscarriage because I wouldn't have to become a parent. But then I felt guilty, so, of course, I stopped.

Frida describes how she went against her own wish of having an abortion or continuing to use tobacco because of a bad conscience against her husband as well as the fetus. She surrenders to the role of a good wife, and the cultural notion of a good mother who puts her baby’s well-being first by not exposing the fetus to unnecessary risks (Lupton 2012). She does not see motherhood as a promise of happiness per se, and is therefore reluctant, but she expects motherhood to have a positive impact on her marriage, which outweighs her doubts.

As we can see, all women describe their decision-making as relational (Madeira 2014), influenced by their partner or significant others (parents, friends, medical professionals). However, in one case it is not just influenced by others, it can even be described as reproductive coercion (Moulton *et al*. 2021) and a result of psychological violence. Jonna described how she had never felt any biological urge to have children, and had also been afraid since her teens of giving birth and becoming a single mother. However, she got involved with a man who turned out to be violent, and eventually manipulated her into motherhood:

When we had lived together for about a year, then he had been on me a lot about having children, and I had gotten pregnant once and had an abortion because I didn't want to have children with him at all. But in the end, I had tried everything I could to make him feel better, because that was what he blamed his violence on, that he didn't feel well. And I was in such a bad state, so I kind of caved to his pressure and started living my life the way he wanted me to live it, and one of those things was that I started to take on his worlds of thought and values. That made it easier for me to live. And part of this is that I finally agreed that we're going to have a child // So we ended up deliberately, sort of, getting me pregnant. It was a conscious choice; I had an IUD^1^ at the time, but I took it out to be able to get pregnant and then I got pregnant quite quickly. I guess I never thought I would survive as long as to see my child. It was a very abstract experience. It was somehow “Now I do this and then he will be happy”, and the thought didn't go much further than that.

The partner did become happier, but it did not last for very long. Soon after birth, he went into a severe depression, continued the psychological violence and left her alone with all the responsibilities for the child. Thus, the nightmare that she had been afraid of since her teens became real.

Just like all the other women, Jonna returned to the fact that she became pregnant ‘consciously’, even though not wanting to become a mother, which invokes feelings of shame and guilt. In their minds, this consciousness led to a moral obligation of not having an abortion or giving up motherhood through adoption. Their reasonings are likely influenced by the the abortion debate that is framed in moral terms—life versus lifestyle—which makes the arguments pro-abortion sound (and feel) much more shallow than pro-life arguments (Madeira 2014, 44).

The women’s stories are also reminiscent of the old debate between nature and culture, where women are seen as closer to nature, and motherhood is seen as a natural and essential part of what it means to be a woman. Being oriented towards motherhood holds a promise of happiness (Ahmed 2010), which the women in my study put their faith in. Also, when their partner is aligned with the idea of the family as a happy object, it becomes harder to avert motherhood. Amanda makes this clear when explaining, ‘*So right from the start, I really regretted it. But I did it anyway, because I still thought that it must be me who’s wrong because everyone else thinks it’s so amazing. And I clung to the fact that it will be a different thing when the baby comes out’*. Hence, they follow the path that so many women before them have trodden (Ahmed 2006), and the temporal logic of ‘linear progress over time’ leads them to think that positive feelings towards motherhood will develop (Donath 2015).

## Dealing with motherhood

Despite their hopes, however, experiencing motherhood did not change their minds. Although parts of parenting were rewarding, they described motherhood as a *prison*, *tunnel*, *treadmill* or an *even greyness*. Leaving the family, however, was not seen as an option. Motherhood was something they had to deal with for the rest of their lives: ‘Now I am a parent until I die, so I just have to close the door; no matter if I want to or not’, Rebecca said. Mothers felt obliged to face the music and try their best to become attached to their children. They read handbooks on parenting and child psychology, used a baby carrier and breast fed to be physically close to the child, and acted in ways that were considered best for the child’s development.

Their experiences of performing these motherhood tasks differed. For some, it was easy to take on the practical tasks. One mother described how the shame of having brought a child into the world that she did not really want motivated her to be very involved, so her child would never notice her regret. Other mothers had a much harder time performing the motherhood tasks, and their husbands have had to step up and take more responsibility. One of them described:

The children have a great relationship with my husband because he probably takes seventy per cent of all the responsibilities at home. I try to take as little responsibility as possible. I do everything one should do; I keep track of doctor appointments; of course, I give them food and water [chuckles] but I don't want to do much more with them. I try to keep my distance // I've been feeling a little unwell [emotionally]. So, I haven't been able to be that active with them, because I just think they're annoying. But I took theatre lessons when younger, so I'm pretty good at acting [chuckles]. So, even though I have not been feeling well, even on the worst days when I just want to run away, I've sat and played with them and talked to them and stuff like that. But you can't keep up a facade for too long either. Sometimes you lose it a bit. Then I get mad [laughs]. My husband, he is very attentive, he notices quite quickly when I start to get irritated, so then he says “Now you should go away and take a break”, and I take five min here and there and go and cry in the bathroom [chuckles].

Although she laughs several times when telling her story, it is not a happy laugh. It is a laugh to make what she is saying bearable to say, instead of starting to cry.

All women had, in one way or another, taken a step back as a mother as a means to handle their distress about motherhood. Kerstin explained how being on maternity leave was not her thing, and she was very happy when she could start working again, saying *‘I didn't go into a depression because I was a mother or anything, but it was, um it wasn't what I was suited for’*. She had seen a clear difference between other people’s ways of being mothers and her own, and described herself as less engaged than a more involved mother in the neighbourhood. The differences between them had continued even after the children had grown up. The neighbour is now highly involved in her grandchildren, while Kerstin felt quite relieved when the children had moved away from home and she could start living as usual again. It is clear that the many happy years Kerstin, and other still married mothers, had had with her partner before having children played a role in how they now experienced regret. They were nostalgic about the past and longed back to the carefree life they had had together before having children. The divorced mothers were also nostalgic, but talked more about their former selves as individuals. Married or not, all women talked about how they missed spontaneity, independence and more options in their lives.

## Conclusion

In this article, I have tried to answer the question of why women who do not long for children still have them despite reproductive choice being technically available to them. The women’s narratives show how complex reproductive decision-making can be, and how it is always made in relation to others (Madeira 2014). They also show how reproductive decision-making is influenced by *the possibility of regret* if one does *not* have children, as well as medical and moral discourses. All these aspects are related to biosocial expectations of womanhood. The women’s stories construct the decision to become a mother as preferably based on a biological and inner urge, which sometimes comes in conflict with expectations of the modern women to be intellectual and rational. A proper woman should be oriented towards motherhood and long for children, and when becoming a mother, put her baby’s needs first and feel blessed by motherhood. Women are expected to be close to nature and guided by its forces (eg, hormones), leading them to procreation and being nurturing. But as humans, women are also expected to be rational, and should therefore be able to make well-considered decisions. This clearly puts women in the middle of the long-lasting conflict of nature versus culture, but also individualism versus collectivism, which creates moral dilemmas.

Motherhood is constructed as *the* object of happiness, and something that a woman does not want to miss out on, even if she does not long for it. It is, therefore, not a surprise that mothers made their decision based on a fear of missing out, or a fear of possible regret. In our neoliberal times, where individuals are imagined as free subjects, women are seen as responsible for their own success as well as failure. As described, the women in this study upheld norms of good motherhood and took moral responsibility through several measures, such as ‘choosing life over lifestyle’ (Madeira 2014) by not having an abortion; remaining in motherhood, instead of giving the child away for adoption or leaving the family; and proving, through their actions, that they were engaged parents who cared for their children and their well-being. Still, they are haunted by shame and guilt, since current ‘feeling rules’ around parenthood (Hochschild 2012) do not allow for too much negativity.

Is it, then, regret that these women were feeling, despite having low expectations of motherhood from the start? I would argue so. More specifically, it is what Lucas (2004) refers to as existential regret, which arises when one feels that one has made a decision against one’s inner wish or gut feeling. It arises, for example, when one has chosen what feels easiest at the time, rather than what one really wants. Some of the women had a hard time dealing with their unwillingness to have children in relation to social pronatalist pressure, which made them take a risk, hoping that they would be wrong and that their maternal instincts would develop in accordance with the logics around motherhood, as described by Donath (2015). All of them turned out to be able to care for their children, carry them close, nurture and protect them, which one of them described as *practical* maternal instinct. What did not happen to them, however, was the emotional or spiritual transformation into a woman who saw motherhood as the meaning of life, as something mythical and natural, or worth it no matter the costs. That they compare their experience with an ideal becomes clear in statements such as *‘It really doesn't feel like I'm meant to be a mom’* or *‘It wasn't was I was suited for’*. Thus, despite being mothers, their fantasies continue to be oriented towards non-motherhood.

To conclude, this study has contributed to a broader understanding of how complex reproductive decisions and related emotions really are, and how the narrative and understanding of these decisions take shape. Even if the influence of others sometimes reflects purely relational conditions (eg, the partner wants to have children), it should nevertheless be understood as shaped within the framework of strongly normative socially, culturally and historically constituted ideals. The examples from this study show a diversity that takes us away from simplistic either-or models, and questions the image of a free liberal subject who independently makes reproductive choices.

## Data Availability

Data sharing is not applicable since the data generated contains hihgly sensitive personal data and falls under the Privacy Act.

## Footnotes

Abbreviation of intrauterine device, a female contraceptive method.
